# Latent Class Analysis in Depression, Including Clinical and Functional Variables: Evidence of a Complex Depressive Subtype in Primary Care in Chile

**DOI:** 10.1155/2021/6629403

**Published:** 2021-02-11

**Authors:** Verónica Vitriol, Alfredo Cancino, Carlos Serrano, Soledad Ballesteros, Marcela Ormazábal, Marcelo Leiva-Bianchi, Carolina Salgado, Cristian Cáceres, Soledad Potthoff, Francisca Orellana, Andrea Asenjo

**Affiliations:** ^1^Medical School, Universidad de Talca, Chile; ^2^Psychology School, Universidad de Talca, Chile; ^3^Mental Health Program, Maule Regional Health Service, Chile; ^4^Medical School Universidad Católica del Maule, Chile; ^5^Instituto de Neurociencias de Viña del Mar and Corporación de Neuropsiquiatría de Santiago NEPSIS, Chile; ^6^Primary Health Care Department, Talca, Chile; ^7^Primary Health Care Department, Curicó, Chile

## Abstract

**Objective:**

To establish differentiated depressive subtypes using a latent class analysis (LCA), including clinical and functional indicators in a sample of depressed patients consulted in Chilean Primary Health Care.

**Methods:**

A LCA was performed on a sample of 297 depressed patients consulted in Chilean PHC. The Mini International Neuropsychiatric Interview, the Hamilton Depression Rating Scale, the Outcome Questionnaire -social role, and interpersonal subscales were as instruments. A regression analysis of the different subtypes with sociodemographic and adverse life experiences was performed.

**Results:**

In a sample characterized by 87.5% of women, two, three, and four latent class models were obtained. The three-class model likely represents the best clinical implications. In this model, the classes were labeled: “complex depression” (CD) (58% of the sample), “recurrent depression” (RD) (34%), and “single depression episode” (SD) (8%). Members of CD showed a higher probability of history of suicide attempts, interpersonal, and social dysfunction. Psychiatric comorbidities differentiated the RD from SD. According to a multinomial regression model, childhood trauma experiences, recent stressful life experiences, and intimate partner violence events were associated with the CD class (*p* < 0.01). *Limitations*. The vast majority of participants were females from Chile and the sample studied was not random. So, the results may not necessarily represent outpatient clinics.

**Conclusions:**

This study can provide additional evidence that depression, specifically in female gender, could be better understood as a complex heterogeneous disorder when clinical and functional indicators are studied. Furthermore, adverse life experiences starting in childhood could lead to a differentiated complex depressive subtype.

## 1. Introduction

Depression is a leading cause of disability worldwide [1]. In Chile, as in the world, this disorder constitutes a significant public health problem [2]. Recent findings show that, in the last year, 18.2% of the adult Chilean population present depressive symptoms and 6.2% meet the criteria for major depression (MD) [3, 4]. It is the second leading cause of disability-adjusted life years (DALYs) and the first one among women between 20 and 44 years [2, 5].

In the Chilean health system, most of MD patients (90%) are diagnosed and treated at primary health care (PHC) [2, 6]. At this level of care, among patients with MD, the prevalence of anxiety comorbidity and adverse life experiences is higher than 80% [7–9], associated with greater symptom severity [10] and lower clinical remission at 12 months [11]. These findings are consistent with the current knowledge in the field, [12–17], which also shows that neither psychiatric comorbidities nor adverse life experiences are adequately screened in PHC clinical samples [18–20].

The importance of anxiety in MD is recognized as a specifier in the DSM-5 [21] and revealed by the need for a specific treatment approach [22]. However, in the current clinical guideline, there is a lack of specific recommendations for MD with adverse life events, specifically childhood trauma experiences (CTEs) [22–25]. Last 30 years of evidence shows that CTEs in MD are characterized by the presence of psychiatric comorbidities, chronicity, suicidal tendencies, refractory to standard treatments [22, 23, 26–28], interpersonal difficulties, and emotional dysregulation [29]. This complex clinical picture could constitute a specific depressive subtype that should be recognized in clinical practice (19.22, 23). Nevertheless, greater knowledge in this area is required.

Latent class analysis (LCA) is a statistical technique that detects different subtypes in apparently homogeneous samples [30, 31]. Most LCAs in MD are based on depressive symptom associations, with no consistent evidence yet [32–35]. Psychiatric comorbidity, suicide, and interpersonal trauma have hardly been used as indicators of LCAs in MD [33]. A recent meta-analysis [33] evidenced that only one LCA included suicide as an indicator [34]. Another study found that anxiety was a distinctive feature within moderate MD [36]. Meanwhile, CTEs have studied more in their associations with specific subtypes of depressive symptoms, with conflicting results [37–39]. Furthermore, there is no evidence of the inclusion of functional variables as indicators of LCAs in MD [33].

The objectives of this research are to establish the presence of depressive subtypes in a Chilean PHC sample, using a LCA including clinical and functional variables as indicators, and then to determine the association between these different subtypes with sociodemographic features and adverse biographical antecedents.

## 2. Methods

### 2.1. Design

This is a cross-sectional study of the data belonging to the research project “Factors associated with the different evolutions presented by patients who entered to treatment through health explicitly guarantees in Depression in PHC: follow-up of a cohort” (Project FONIS SA13/20135). The protocol was approved by the Ethics and Research Committees of the University of Talca (2013-080) and the Maule Regional Health Service.

### 2.2. Sampling and Methods

In the original project, an intentional sample of 334 patients was calculated using the SPSS version15 program, considering an outcome difference of 40% [40], a dropout rate of 30%, and a follow-up of 20% [41].

Inclusion criteria were age older than 15 years, having entered to treatment for depression by the primary care team, being able to give and sign an informed consent, and meeting the criteria for MD according to the Mini International Neuropsychiatric Interview (MINI) [42].

Exclusion criteria were organic brain damage, sensory disabilities, and immediate referral to secondary health care services due to current suicide attempts, bipolarity, and/or psychosis.

Forty-four patients were referred by the primary care team to the study. After giving and signing the informed consent, they were interviewed by a team comprising psychiatrists and psychologists with over 10 years of experience. Three hundred ninety-four patients met the criteria for MD. Two hundred ninety-seven patients completed the assessments and were included in the analysis of this study.

### 2.3. Instrument

#### 2.3.1. Semistructured Clinical Interview, Designed by the Team

It gathered sociodemographic background data (age of consultation, marital status, schooling level, and current work activity) and clinical history of depressive illness (including previous depressive episodes and/or treatment for a previous depressive episode).

#### 2.3.2. Screening for CTEs [43]

This clinician-administered instrument evaluates memories of adverse experiences occurring before age 15: traumatic separation from the father, mother, or caregiver for more than one month; abuse of alcohol or drugs by a family member; physical abuse; physical harm associated with punishment; physical violence between parents or caregivers; forced sexual contact by a relative or nonrelative. The external validity of this screening has been confirmed [44], obtaining a Pearson's correlation coefficient of 0.88.

#### 2.3.3. Intimate Partner Violence Events (IPVEs) Questionnaire

It consists of items on physical, psychological, economic, and sexual violence. This scale was validated in Spanish with a Cronbach alpha 0.91, specificity of 94% and a sensitivity of 89% [45].

#### 2.3.4. The Life Experiences Survey (LES)

It is a clinician-administered instrument developed by Sarason [46], translated and validated in Spanish with a Cronbach alpha 0,7 and construct validity ≥0.50 [47]. It consists of 47 items that investigate stressful life experiences (LEs) during the previous six months with both positive and negative connotations. For this study, only negative events were considered.

#### 2.3.5. The Mini International Neuropsychiatric Interview (MINI) [42]

It is a clinician-administered diagnostic interview with a kappa coefficient of 0.69, a sensitivity of 89%, and a specificity of 92% [48]. This brief and structured interview assesses the presence of the main psychiatric disorders of the DSM-IV and ICD-10. It is divided into modules identified by letters, each corresponding to a diagnostic category. In our case, the research team used the Spanish version [42], which excludes the psychosis module.

The suicide module of the MINI (MINI-C) investigates suicidal tendencies in the last month, including dichotomous answers to five questions:

C1: Have you thought you would be better off dead, or have you wished you were dead?

C2: Did you want to hurt yourself? C3: Have you thought about suicide?

C4: Have you planned how to commit suicide?

C5: Have you tried to commit suicide?

In addition, it is included the C6 question, which asks about suicidality during life:

C6: Throughout your life, have you ever attempted suicide?

According to the research protocol, patients who obtained positive results in C4 and/or C5 were excluded from the investigation and referred to the secondary health care level.

#### 2.3.6. The Outcome Questionnaire (OQ45-2), Designed by Lambert [49]

It is a self-administered instrument evaluating symptom distress, interpersonal relations, and social role. Validated in Chile with psychometric properties: sensitivity 0.9 and specificity 0.93 [50]. For the purposes of this research, the interpersonal and social subscales were used. The interpersonal subscale includes 12 questions and scores of 16 points or more indicate interpersonal dysfunction. The social role subscale includes 9 questions and scores of 14 points or more indicate social role dysfunction [49]. It was used at baseline, six and twelve months.

#### 2.3.7. Hamilton Depression Rating Scale, 17 Items (HDRS-17), Used at Baseline, Three, Six, Nine, and Twelve Months

This scale determines the severity of depression symptoms. Validated in Spanish [51] with Cronbach alpha ≥0.7, intraclass correlation coefficient (ICC) ≥0.9, interrater reliability (ICC) ≥0.9, and sensitivity to change (effect size) ≥1.5 [51]. The National Institute for Health and Care Excellence (NICE) recommends 18 points or less for mild and moderate cases and 19 points or more for severe cases [52].

### 2.4. Statistical Analysis

An LCA was performed to assign patients to different nonapparent subpopulations, using the Mplus 7.0 program. This clustering approach consists of a probabilistic model that describes the distribution of the data and assesses the probability that certain cases are members of certain latent classes [53].

Depression severity (19 or more points in HDRS), suicidal thoughts (positive answers in MINI C1-3), past suicide attempts (positive answers in MINI C6), psychiatric hypercomorbidity (3 or more in MINI comorbidities), course (single or recurrent episodes), interpersonal dysfunction (16 or more points in OQ45-2 interpersonal subscale), and social role dysfunction (14 or more points in OQ45-2 social role subscale) were the indicators included in this LCA.

The model fit for different number of classes was done through the log-likelihood and Expectation-Maximization (EM) algorithm [54], considering that LCA was optimal when the classes were as homogeneous as possible and the differences between classes were as large as possible [53]. Subsequently, the probability of membership in each latent class and the conditional probability of response to the item were established. The models were compared according to three criteria that consider goodness of fit and parsimony: the Bayesian Information Criterion (BIC), the Akaike Information Criterion (AIC), and the Adjusted Bayesian Information Criterion (Adjusted BIC) (Nylund & Muthén, 2011). In these, lower values indicated a better fit of the model. The inclusion of another class to the model was considered when the Lo-Mendel-Rubin Test (LMR) and the Bootstrap Likelihood Ratio Test (BLRT) were significant [53].

Finally, a multinomial regression model was performed between the different subtypes and sociodemographic features and adverse life experiences (CTEs, LEs, IPVEs) using the Python (stats model).

## 3. Results

Table 1 shows the sample's characteristics. It is important to note that 87.5% of the sample are women.

Table 2 displays the models. In this case, the two-, three-, and four-class models have a good fit, that is, the AIC, BIC, and the adjusted BIC values are significantly lower than the ones for the five-class model. Likewise, the LMR (*p* < 0.02) and the BLRT (*p* < 0.01) show that the three-class model is surpassed by a four-class model. In the same way, a high degree of differentiation between the three-class model (entropy = 0.77) and the four-class model (entropy = 0.82) is obtained, but not among subjects belonging to the same class.

Table 3 summarizes the probability that a patient will respond affirmatively to an item, as a function of its inclusion in one of the models. In all models, we found the following: first, a higher probability of a history of suicide attempt goes hand in hand with a higher probability of social and interpersonal dysfunction. Second, patients with a higher probability of psychiatric comorbidities have a higher likelihood of recurrence. Finally, patients who have a greater probability of more severe symptoms are more likely to have suicidal thoughts.

If we are considering the clinical and functional variables as indicators of different subtypes, the three-class model exhibits the best clinical implications.

In this model, the classes obtained are labeled as follows:

- Complex depression (CD), characterized by recurrence and high probability of psychiatric comorbidities, history of suicide attempts, and interpersonal and social difficulties
- Recurrent depression (RD), characterized by recurrence, high probability of psychiatric comorbidity and low probability of history of suicide attempts, and interpersonal and social difficulties
- Single episode depression (SD), characterized by a single episode and low probability of psychiatric comorbidity, history of suicide attempts, and interpersonal and social dysfunctions

Finally, the multinomial regression model (Table 4) shows a significant association of CTEs (*B* = 0.18; *p* < 0.01), LEs (*B* = 0.17; *p* < 0.02), and IPVEs (*B* = 0.05; *p* < 0.003) with the CD class, compared to RD class. Likewise, it is possible to observe a significant association of CTEs (*B* = 0.24, *p* < 0.04), LEs (*B* = 0.28, *p* < 0.03), and IPVEs (*B* = 0.08, *p* < 0.02) with CD class, compared to the SD class. There are no significant differences in these variables when RD and SD classes are compared.

## 4. Discussion

To our knowledge, this is the first study that includes clinical and functional indicators in a LCA performed in a PHC clinical depression sample. It is important to note that almost 90% of this sample corresponds to women. The results obtained, in this case in a predominantly female sample, provide additional evidence that depression could be best conceptualized as a complex heterogeneous disorder when clinical and functional variables are considered [55–60].

The inclusion of functional variables in the LCA of this study evidenced a depressed patient profile related to psychosocial factors. Likewise, a LCA study performed in patients with posttraumatic stress disorder (PTSD) [61] revealed a more complex PTSD characterized by the presence of interpersonal difficulties, affective dysregulation, and alterations in self-esteem, associated with exposure to interpersonal adversities, particularly CTEs [62]. In another type of clinical sample, a data-driven study that explored the heterogeneity of resources in cancer and psoriatic patients allowed the identification of different patient profiles, associated with different intensity of posttraumatic growth [63]. In all these researches, the data-based approach distinguished relevant clinical subtypes, in which psychosocial and functional variables explain the need for patient-centered recognition, beyond the standardized diagnosis.

Specifically in this research, the statistical methodology shows that the four-class model presented the best statistical solution. However, the three-class model, which also has a good fit, might represent the best clinical implication. In this model, each subtype (CD, RD, and SD) requires the recognition of clinical and functional variables that are underdiagnosed in clinical samples. In the four-class model, the other new class arises from the CD class in patients with less severe symptoms at the moment of assessment.

The CD class evidenced in this study presented the characteristics that the literature describes in patients with MD and interpersonal trauma: suicidality, chronicity, interpersonal, and social dysfunction [22–29]. This complex clinical picture is understood through the neurobiological and psychological sequelae generated by exposure to prolonged adversity, mainly since childhood [23, 24, 61, 62, 64]. This result could be providing more evidence about the existence of a specific MD subtype associated with adverse interpersonal life experience. A recent study, using a data-driven approach, identified a neurophysiological depressive subtype related to CTEs and dysfunction in specific neural circuits [65].

The clinical implications, if this CD profile could be corroborated in other clinical samples, are the following: first, CTEs and other adverse life experiences are poorly recognized in MD clinical samples [66, 67]. Second, patients with depression and CTEs are often refractory to standard treatments [27, 28] and need a differential treatment approach [68, 69]. Third, there is a lack of recommendations in current clinical guidelines on the importance of recognizing the interpersonal, social, and emotional consequences of patients with MD and interpersonal adversity [70]. Fourth, health care teams may need to be trained in the trauma-informed care model [71, 72]. This paradigm points to a deep understanding of the impact of trauma on adult health, and one of its fundamental principles is to avoid revictimization when these subjects consult seeking help [73].

A last clinical implication of this research is the relationship between psychiatric comorbidity and recurrence. Most of the psychiatric comorbidities observed in this population correspond to the anxious spectrum [8, 9]. The importance of anxiety in depression is recognized in the DSM-5 [21, 57, 58]. However, according to current knowledge, anxiety in depression is unrecognized in clinical practice, specifically in PHC settings [18]. This lack of recognition is associated with chronicity and requires differentiated pharmacological and psychotherapeutic treatment [22].

Among the limitations of this study, it is important to note that the sample studied is not random and also does not include a control sample, so the results may not necessarily represent all depression patients, from PHC or from other outpatient clinics. The prevalence of the different subtypes might be explained by an overrepresentation of female patients with a more severe and complex clinical picture, probably due to the recruitment process, as providers may have referred to the study the most seriously ill patients, who agreed to participate. It is interesting to note that the frequency of traumatic exposure in this sample confirms previous studies in Chile [19].

Another limitation is that the CTES detection tool used in this study does not include emotional abuse, which is the CTE most clearly associated with MD [14]. It is not possible to make inferences about causality between adverse life experiences and depressive subtypes nor about the influence of depression on a higher tendency to report childhood trauma events. A control sample should be incorporated in further studies.

Regarding the strengths of this work, as far as we know, this is the first study to perform a LCA in a Latin American sample of PHC depressed patients, which simultaneously incorporates the assessment of clinical and functional characteristics, using instruments validated in Chile. The use of a data-driven approach gives more evidence on the importance of including functional and psychosocial variables in search of different clinical profiles from a patient-centered perspective.

## 5. Conclusion

This study provides evidence that MD, specifically in the female gender, can be better understood as a heterogeneous disorder of multiple subtypes, in which clinical and functional features that are underrecognized in clinical practice should be considered. It also provides evidence that interpersonal adversity across the lifespan could lead to a distinct depressive subtype.

It is necessary to replicate this study in other depressive populations, including additional functional variables, to ratify and give more consistency to the findings obtained in this research.

## Figures and Tables

**Table 1:** Sociodemographic, clinical, and psychosocial characteristics in 297 Chilean PHC patients.

Sociodemographic characteristics		

Sex	*N*	%
Women	260	87,5

Scholarship	*N*	%
Less than high school	118	39.7
High school/partial high school	123	41.4
College/partial college	56	18.9

Other features	*N*	%
Lives with partner	135	45.4
Lives alone	36	12.2
Employed with income	39	13.2

	Mean	Standard deviation
Age	47,5	15

Clinical characteristics of depression		

	Mean	Standard deviation
Age at the first episode (years)	30.7	17.2
Number of depressive episodes	3.64	4.2
Duration of the longest episode (years)	3.58	7.2
Depressive symptoms at baseline HDRS (points)	20	4.6

	*N*	%
History of suicide attempt	96	32.3
History of previous depression treatment	94	31.6

Comorbidities		

Biomedical	*N*	%
0	133	44.8
1	64	21.4
3	56	18,8
More than 3	44	15.0

Psychiatric	*N*	%
0	47	15,8
1	58	19.5
2	57	19.1
3	54	18.1
More than 3	81	27.2

Adverse life experiences		

IPVEs intimate partner violence events (entire adult life)	*N*	%
0	124	41.8
1	19	6.3
2	14	4.7
3 or more	140	47.2

CTEs childhood trauma experiences	*N*	%
0	53	18.0
1	48	16.2
2	50	16.8
3	49	16.4
4 or more	97	32.6

LEs life adverse experiences prior six months		

0	23	7.6
1	63	21.1
2	65	22.1
3	48	16.2.
4 or more	98	33.0

**Table 2:** Latent class analysis models in 297 Chilean PHC depression patients.

No. of classes	AIC	BIC	Adjusted BIC	LMR *p* value	BLRT *p* value	Entropy
2	2892.99	2955.78	2901.87	<0.01	<0.001	0.584
3	2875.09	2971.12	2888.67	0.03	<0.001	0.733
4	2864.69	2993.97	2882.79	0.02	<0.001	0.818
5	2871.85	3034.38	2894.84	0.09	.10.000	0.720

**Table 3:** Likelihood of each indicator according the two, three, and four-class models, in 297 patients in primary care in Chile.

Model	Hamilton	Previous suicide attempt	Suicidal thoughts	Psychiatric comorbidities	Previous depressive episode	Interpersonal dysfunction	Social dysfunction	*N* (%)
*2-class model*								
CD^∗^	**0.676**	**0.509**	**0.729**	**0.974**	**0.829**	**0.809**	**0.613**	150 (51)
RD^∗∗^	0.363	0.159	0.237	0.698	0.533	0.285	0.305	147 (49)
*3-class model*								
CD^∗^	**0.663**	**0.474**	**0.712**	**0.965**	**0-813**	**0.778**	**0.592**	174 (58)
SD^∗∗∗^	**1.000**	0.043	**0.528**	0.170	0.000	0.251	0.363	23 (8)
RD^∗∗^	0.207	0.176	0.124	**0.766**	**0.610**	0.251	0.273	100 (34)
*4-class model*								
CD^∗^	**0.712**	**0.497**	**1.000**	**0.977**	**0.779**	**0.783**	**0.541**	124 (41)
CD (with less suicide thoughts)	0.474	**0.389**	0.000	**0.858**	**0.907**	**0.746**	**0.822**	53 (18)
SD^∗∗∗^	**1.000**	0.043	**0.508**	0.111	0.000	0.250	0.375	20 (7)
RD^∗∗^	0.241	0.181	0.135	**0.800**	**0.588**	0.245	0.211	100 (34)

^∗^Complex depression (CD) (high likelihood of previous suicide attempt, interpersonal and social dysfunctions, psychiatric comorbidities, and recurrence). ^∗∗^Recurrent depression (RD) (high likelihood of psychiatric comorbidities and recurrence). ^∗∗∗^Single depression (low likelihood of previous suicide attempt, interpersonal, social dysfunction, psychiatric comorbidities, and recurrence).

**Table 4:** Multinomial regression model between complex depression (CD), recurrent depression (RD), single depression (SD) classes and on sociodemographic and adverse biographical background (CTEs, LEs, IPVEs), in 297 Chilean PHC depression patients.

	CD/RD					CD/SD					RD/SD				
	B	SE	*z*	*p*	C I (0.025-0.975)	B	SE	*Z*	*p*	CI (0.025-0.975)	B	SE	*Z*	*p*	CI
Constant	-1.5	0.76	-1.96	0.05	-3—0.001	-0.05	1.32	-0.38	0.7	.3.03--2.09	-0.7	1.16	-0-60	0.54	-2.97—1.56
Age	0.02	0.01	0.29	0.77	-0.02—0.023	-.0.01	0.017	-1.02	0.3	-0.06—0.01	-0.015	0.02	0.93	0.35	-0.07—0.047
Sex	0.54	0.41	1.29	0.19	-0.02—1.63	0.66	0.63	1.05	0.3	-0.57—1.9	-0.19	0.53	-0.36	0.71	-1.25-0.86
Educational level	0.09	0.08	1.18	0.23	-0.06—0.25	0.20	0.13	1.49	0.1	-0.06—0.46	-0.17	0.13	-1.3	0.19	-0.43—0.88
Live with partner	-0.19	0.11	-1.65	0.098	-0-41—0.035	-0.02	.0.17	-0.13	0.9	-0.36—0.31	-0.13	0.19	-0.67	0.5	0-49—0.24
CTEs	**0.18**	**0.73**	**2.49**	**0.01**	**0.038-0.32**	**0.24**	**0.11**	**2.03**	**0.04**	**0.009-0.47**	-0.06	0.12	-0.5	0.61	-3-06-0.18
LEs	**0.17**	**0.73**	**2.30**	**0.02**	**0.025—0.311**	**0.28**	**0.13**	**2.10**	**0.03**	**0.047—0,54**	-0.10	0.13	-0.75	0.45	-3.66—0.16
IPVEs	**0.05**	**0.02**	**2.92**	**0.02**	**0.017—0.087**	**0.07**	**0.03**	**2.42**	**0.01**	**0.015—0.14**	-0.01	0.03	-0.45	0.65	-0.081—0.51

CTEs: childhood trauma experiences; LEs: life experiences; IPVEs: intimate partner violence events. CD: complex depression; RD: recurrent depression; SD: single episode depression. In bold: statistical significant association.

## Data Availability

The data used to support the findings of this study are restricted by the Ethics Committee Universidad de Talca in order to protect patient privacy. Data are available from Dr Veronica Vitriol G mail vvitriol@utalca.cl for researchers who meet the criteria for access to confidential data. The data used to support the findings of this study are available from the corresponding author upon request.
